# Some Farther Thoughts on the Uses of “Phylotacca Decandra.”

**Published:** 1874-02

**Authors:** C. H. Tidd

**Affiliations:** Middleport, Ohio


### SOME FARTHER THOUGHTS ON THE USES OF "PHYLOTACCA DECANDRA."

RY C. H. TIDD, M.D., MIDDLEPORT, OHIO.

In the "*Clinic*, November 29, 1873, I published a short article on the uses of the above drug, giving a report of a few cases in which I had used it, as well as collating the experience of others. Since then I have used it in quite a number of cases, a resume of which, together with such items as I have been enabled to gather from my brother practitioners of this place, shall form the basis of this paper. My object in calling the attention of the profession to this drug is two fold. The first is, "That should it prove as efficacious in the hands of others, as in mine, they will find in it an invaluable agent in the treatment of certain diseases, which have been hitherto found troublesome to treat, and the second is from a sense of duty which I apprehend every physician owes to his fellows namely: to give them the advantage of his experience, so far as it may prove of practical value."

Case 1. In October last Mr. D. called at my office, stating that he was suffering from rheumatism, which affected his right leg. He also stated that he had been a sufferer in this respect for about seven years. I immediately placed him upon an alcoholic extract of "Phytolacca," ordering him to take a dose equaling about three grains, "ter in die." This he continued for about four weeks, when feeling no signs of the former trouble, he discontinued its use, since which time he has felt no return of the disease, although he has constantly been exposed in all kinds of weather.

In November, 1872, Mrs. N., æ 52, mulatto, large, fleshy but active and a hard worker, was found suffering intensely from muscular rheumatism. The pulse was rapid and full, skin hot and dry, tongue furred heavily, no appetite, bowels costive, urine high colored; not tested. She had been in this state for months, but not to the suffering now present. Had taken various remedies with very temporary relief. A saline cathartic was administered, and that followed by an opiate to relieve the extreme pain. She was also placed on the saturated tincture of Phytolacca Decandra; dose fifteen drop ter die, to be gradually increased to the point of producing nausea, and then continued. Nothing else was given. She continued the remedy till one ounce was taken, when she felt so well that the remedy was omitted entirely. She was not seen again till January 18, 1874, when she was apparently in perfect health, and stated that she had not been so free from pain for many months. The muscular pain effected the arms, legs and back, and often the abdominal muscles, frequently becoming so severe that she was compelled to take to her bed.

Case 2. Mrs. B. has suffered every winter for six years from rheumatism affecting her left arm and wrist to such an extent that she was unable to sweep, mix bread, or do any work in fact, which required the flexion or extension of the forearm. She was placed upon twenty-two minum doses of "fl. ext. phytolacca," to be taken three times daily, and after continuing the remedy for three weeks, was entirely relieved, so that at this time, now nearly three months since the discontinuance of the medicine, she is able to perform all the duties requisite on housekeeping, and says she has not felt a single symptom of the return of the disease.

Case 3. Miss E. J. was affected with adenitis, affecting the glands of the post cervical chain, as well as the subnmxillary and sublingual. I could elicit no syphilitic history, consequently placed her on the tincture phytolacca, and in much less time than I had anticipated, was rewarded by seeing a gradual declination of the affected parts, which continued until they had resumed their natural size. In this patient the appetite was much improved and she became fleshier than for several years past, and the improvement still continues.

Dr. T. C. Smith has kindly furnished me the history of the following cases. Mrs. O. at her last confinement but one, had a quite severe attack of phlegmasia dolens, accompanied with mastitis, which in spite of all remedies, came near ending her life. She finally recovered however, although very slowly, and subsequently became pregnant again; after parturition was accomplished she again presented symptoms of a recurrence of her former troubles, in fact the breast became quite hard and nodulous. She was immediately placed upon phytolacca, in as large doses as she could bear, and with the effect of a complete subsidence of the symptoms, which gave her no farther trouble.

Mrs. B. who had been troubled for a considerable time with eruptions of a pustulatory character, was placed upon the tincture phytolacca, under the use of which she made a rapid recovery. This agent has also proved of great benefit, and has acted with promptness, in several cases of acute rheumatism, in the Doctor's practice.

Dr. Bishop, of this place, informs me that with twenty drop doses "*ter. in die*" of the phytolacca, he has recently prevented two cases of threatened mastitis, and has also used it with marked success in some cases of scrofulous eruptions.

That this remedy is equal to potassi iodidii, in all cases of adinitis, I do not claim, but that in *certain cases* it has proven itself vastly superior in my hands I WELL know, and as a remedy in muscular rheumatism, and in some cases of articular, it possesses advantages over many of the highly vaunted remedies of the day.

In conclusion, I would ask of my brother practitioners under whose notice this may come, to give the remedy a trial, take notes of the cases, and report their results to the profession, that we may know whether their experience confirms our testimony.

